# Bacterial Cellulose Hydrogel Incorporating Silver Nanoparticles: A Nanobiotechnological Approach for Skin Infections Caused by MRSA and MRSE

**DOI:** 10.3390/ph19030409

**Published:** 2026-03-02

**Authors:** David N. Oliveira, Lara L. Oliveira, Hanne L. R. Q. Macedo, Yolice P. M. Ruiz, André Galembeck, Danilo E. Xavier, José L. A. Aguiar, Luís A. A. Campos, Isabella M. F. Cavalcanti

**Affiliations:** 1 Keizo Asami Institute (iLIKA), Federal University of Pernambuco (UFPE), Av. Prof. Moraes Rego, 1235, Cidade Universitária, Recife 50670-901, Pernambuco, Brazil; david.dno@ufpe.br (D.N.O.);; 2 Department of Fundamental Chemistry, Federal University of Pernambuco (UFPE), Av. Jorn. Aníbal Fernandes, Cidade Universitária, Recife 50740-560, Pernambuco, Brazil; 3 Northeast Center for Strategic Technologies (CETENE), Recife 50740-545, Pernambuco, Brazil; 4 Aggeu Magalhães Institute, Oswaldo Cruz Foundation Pernambuco (Fiocruz/PE), Recife 50740-465, Pernambuco, Brazil; 5 Department of Surgery, Federal University of Pernambuco (UFPE), Recife 50670-901, Pernambuco, Brazil; 6 Laboratory for Teaching, Research and Extension in Microbiology and Parasitology (LEPEMP), Ouricuri Campus, University of Pernambuco, Ouricuri 56200-000, Pernambuco, Brazil; 7 Laboratory of Microbiology and Immunology, Academic Center of Vitória (CAV), Federal University of Pernambuco (UFPE), Vitória de Santo Antão 55608-680, Pernambuco, Brazil

**Keywords:** silver, antimicrobial resistance, nanostructures, hydrogel, antibiotics

## Abstract

**Background**: Healthcare-associated infections (HAIs) caused by biofilm-forming *Staphylococcus aureus* and *Staphylococcus epidermidis* represent a major public health challenge due to their high resistance and involvement in skin, wound, and soft-tissue infections. In this context, silver nanoparticles (AgNPs) incorporated into *Gluconacetobacter* sp. bacterial cellulose hydrogel emerge as a promising alternative therapeutic strategy. **Methods**: AgNPs and hydrogels were synthesized and characterized using physicochemical and morphological analyses. Antibacterial activity was assessed by determining the minimum inhibitory concentration (MIC) and minimum bactericidal concentration (MBC) following CLSI guidelines, as well as by time–kill curve assays. Antibiofilm activity was evaluated through the determination of minimum biofilm inhibitory concentration (MBIC) and minimum biofilm eradication concentration (MBEC) using crystal violet staining, complemented by scanning electron microscopy (SEM) and Congo red agar method. **Results**: The hydrogel exhibited a three-dimensional microfibrillar structure characteristic of bacterial cellulose, while AgNPs showed rod-shaped, oval, and triangular morphologies, with particle sizes of 35 and 59 nm and positive zeta potentials. MIC and MBC values ranged from 6.25 to 50 µg/mL across all tested formulations and strains. Time–kill assays demonstrated significant bacterial population reductions after 6 to 9 h of exposure. MBIC values ranged from 0.78 to 50 µg/mL, whereas MBEC values ranged from 1.56 to >100 µg/mL. SEM analyses confirmed biofilm disruption, cell eradication, and a reduction in extracellular polysaccharides, particularly for AgNPs incorporated into the hydrogel. **Conclusions**: Overall, the results highlight the strong antibacterial and enhanced antibiofilm potential of AgNP-loaded bacterial cellulose hydrogel against *S. aureus* and *S. epidermidis*, supporting its potential application in infection treatment.

## 1. Introduction

Healthcare-associated infections (HAIs) are acquired during the process of care in hospitals or other healthcare facilities and are not present or incubating at the time of patient admission [1]. According to the World Health Organization (WHO), out of every 100 hospitalized patients, seven in developed countries and ten in developing countries will acquire a healthcare-associated infection, placing a substantial burden on healthcare systems [2].

HAIs cause approximately 40,000 deaths per year, with infection incidence rates reaching up to 25% in developing countries and 15% in developed nations [3]. In such cases, hospital stays are, on average, three times longer, leading to increased direct treatment costs, and higher morbidity and mortality rates [4,5]. Thus, HAIs represent a growing public health concern, particularly when caused by resistant and biofilm-producing bacteria.

According to the WHO, approximately 2000 people worldwide die each day due to resistant infections, a number that could rise to 10 million deaths per year by 2050 [6]. In addition to bacterial resistance, HAIs are strongly associated with the presence of bacterial biofilms, which enable colonization with enhanced structural stability and longevity, forming an impermeable protective barrier that contributes to antibiotic tolerance and/or resistance, and to the persistence of infections, resulting in patient suffering and decreased quality of life [2,7].

In this context, HAIs such as skin, wound, and soft tissue infections caused by antibiotic-resistant and biofilm-producing bacteria, particularly strains of *Staphylococcus aureus* and *Staphylococcus epidermidis*, constitute a significant challenge due to the lack of effective therapeutic strategies to treat these infections, which contribute to the high rates of morbidity and mortality [8,9]. Thus, incorporating a nanostructure into a vehicle, such as a hydrogel with antimicrobial and antibiofilm potential, may promote wound healing and re-epithelialization, representing a promising approach for the effective treatment of such infections.

Given this scenario, the development and application of silver nanoparticles (AgNPs) appear promising due to their well-documented antimicrobial and antibiofilm properties against resistant and biofilm-forming bacteria. However, to treat wound-associated infections effectively, it is essential to employ a topical delivery system capable of efficiently delivering AgNPs [10,11].

In this regard, a bacterial cellulose (BC) hydrogel produced by *Gluconacetobacter* sp. from sugarcane molasses has been explored in various medical fields as a potential therapeutic approach, owing to the presence of polymerized sugars with non-toxic, biocompatible, and tissue-remodeling properties. Moreover, this hydrogel exhibits desirable characteristics for biomedical applications, including high purity, biodegradability, elasticity, flexibility, high porosity, and water-retention capacity [12].

Therefore, the use of silver nanoparticles incorporated into a hydrogel composed of a BC-based biopolymer represents an innovative and promising therapeutic strategy for the treatment of wounds infected by *S. aureus* and *S. epidermidis* strains. Since the bacterial cellulose hydrogel enables the incorporation and topical delivery of metallic nanoparticles, it represents a promising therapeutic alternative against resistant Gram-positive bacteria involved in cutaneous infections, whether in their planktonic form or organized as biofilms. Accordingly, this study evaluated the antibacterial and antibiofilm activities of isolated AgNPs and AgNPs incorporated into a bacterial cellulose hydrogel against resistant and biofilm-producing *S. aureus* and *S. epidermidis* strains.

## 2. Results

### 2.1. Characterization of AgNPs and BC Hydrogel

The AgNPs were characterized based on particle size, zeta potential, and electrophoretic mobility, with particle sizes ranging from 35 to 59 nm, zeta potential values of +32 to +36 mV, and electrophoretic mobilities of 2.56 µS/V/cm and 2.86 µS/V/cm (Table 1).

These characteristics contribute to antimicrobial and antibiofilm activity, as the optimal particle size enhances bacterial cell penetration while the surface charge promotes interaction with the cell membrane.

**Figure 1:**
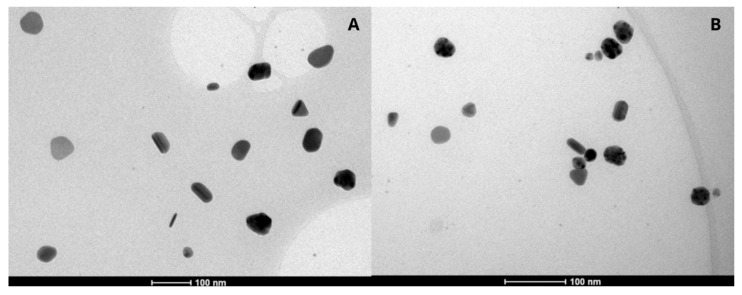
Transmission electron microscopy AgNPs. (**A**) AgNP-C250; (**B**) AgNP-A1000.

The AgNPs were analyzed by transmission electron microscopy (TEM), revealing three predominant shapes: rod, oval, and triangular (Figure 1A,B).

**Figure 2:**
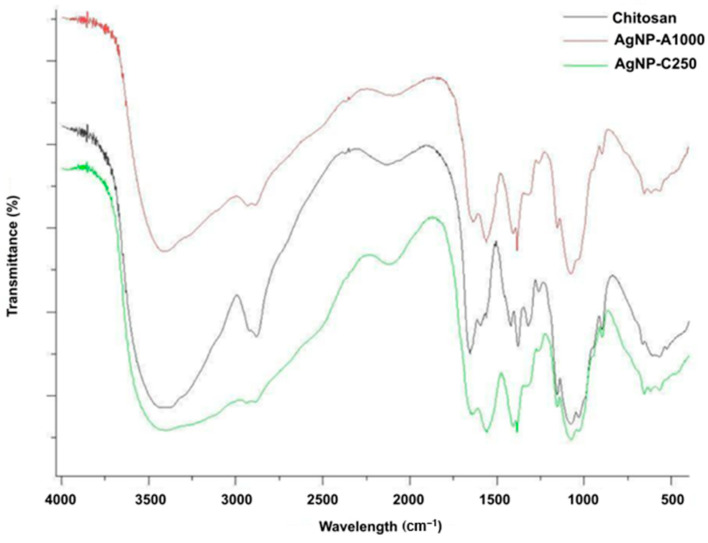
FTIR of silver nanoparticles.

In the FTIR analysis (Figure 2), the presence of chitosan (CH) used in the preparation of the AgNPs is observed, as it stabilizes the system. The FTIR spectra show that CH exhibits intense bands corresponding to O–H/N–H, amide I and II, C–H, C–O–C, and C–O groups, while in the AgNPs these bands are reduced or shifted, indicating interactions between the amino and hydroxyl groups and the silver surface. The changes in the regions ~3400–3200, ~1650, ~1550, ~1400–1370, and ~1080–1030 cm^−1^ confirm the occurrence of coordinated bonds and electrostatic interactions. Additionally, new peaks appear below 800 cm^−1^, which are characteristic of the formation of metallic nanoparticles.

**Figure 3:**
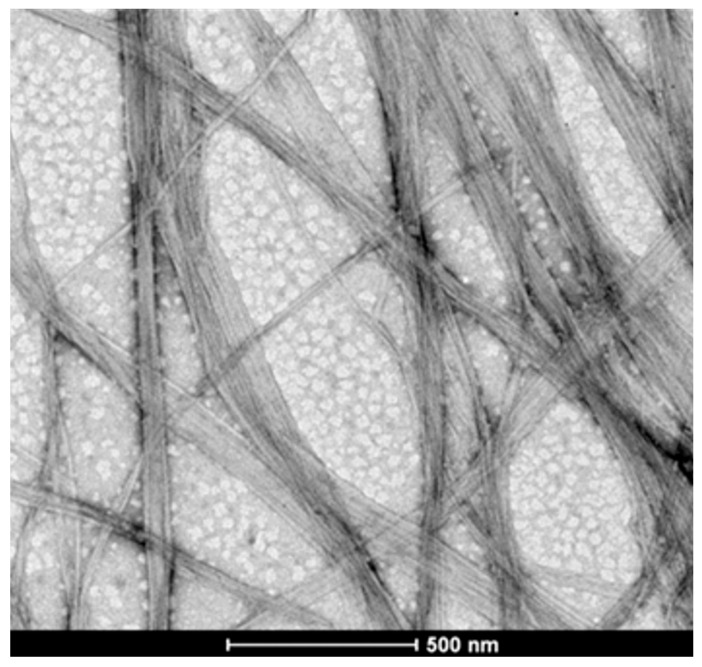
Scanning electron micrographs of bacterial cellulose hydrogel nanofibers.

The hydrogels without incorporated formulations were prepared, and their morphological characterization was carried out using a scanning electron microscope (SEM). The SEM micrographs of the hydrogels are shown in Figure 3. The surface of the gels displays a three-dimensional microfibrillar network, which is characteristic of bacterial cellulose hydrogels.

### 2.2. Evaluation of Antibacterial Activity

AgNP-A1000, AgNP-C250, and AgNP-A1000 incorporated into bacterial cellulose hydrogel (HG-AgNP-A1000), and AgNP-C250 incorporated into bacterial cellulose hydrogel (HG-AgNP-C250) exhibited MIC ranging from 25 to 50 μg/mL against *S. aureus*. AgNP-A1000, AgNP-C250, HG-AgNP-A1000, and HG-AgNP-C250 also demonstrated bactericidal activity, with MBC ranging from 25 to 50 μg/mL against *S. aureus* strains (Table 2).

AgNP-A1000, AgNP-C250, HG-AgNP-A1000, and HG-AgNP-C250 exhibited MICs ranging from 6.25 to 50 μg/mL against *S. epidermidis*. AgNP-A1000, AgNP-C250, HG-AgNP-A1000, and HG-AgNP-C250 also demonstrated bactericidal activity, with MBCs ranging from 25 to 50 μg/mL against *S. epidermidis* strains (Table 3).

### 2.3. Time–Kill Assay

**Figure 4:**
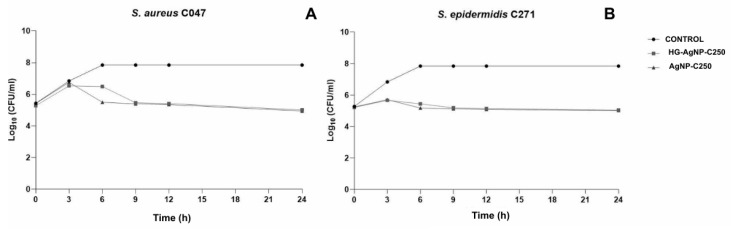
Time–kill curve assay of *S. aureus* and *S. epidermidis* after treatment with AgNP-C250 incorporated into the hydrogel. (**A**) Time–kill curve assay of *S. aureus.* (**B**) Time–kill curve assay of *S. epidermidis*.

The time–kill curves for the clinical MRSA C047 and MRSE C271 strains showed similar time–kill patterns (Figure 4A,B). Both strains exhibited growth inhibition compared to the control group. For both strains, exposure to AgNP-C250 resulted in a reduction after 6 h, while treatment with HG-AgNP-C250 showed a reduction starting at 9 h. After these time points, the number of bacterial cells continued to decrease in terms of CFU over time, with similar values observed for both AgNPs and HG-AgNP-C250.

### 2.4. Evaluation of Antibiofilm Activity

AgNP-A1000, AgNP-C250, HG-AgNP-A1000, and HG-AgNP-C250 exhibited MBIC ranging from 1.56 to 50 μg/mL against *S. aureus* strains. AgNP-A1000, AgNP-C250, HG-AgNP-A1000, and HG-AgNP-C250 also demonstrated MBEC ranging from 1.56 to >100 μg/mL against *S. aureus* strains (Table 4).

AgNP-A1000, AgNP-C250, HG-AgNP-A1000, and HG-AgNP-C250 exhibited MBIC ranging from 0.78 to 50 μg/mL against *S. epidermidis* strains. AgNP-A1000, AgNP-C250, HG-AgNP-A1000, and HG-AgNP-C250 also demonstrated MBEC values ranging from 25 to 50 μg/mL against *S. epidermidis* strains (Table 5).

### 2.5. Evaluation of the Antibiofilm Activity by the Congo Red Agar Method

**Figure 5:**
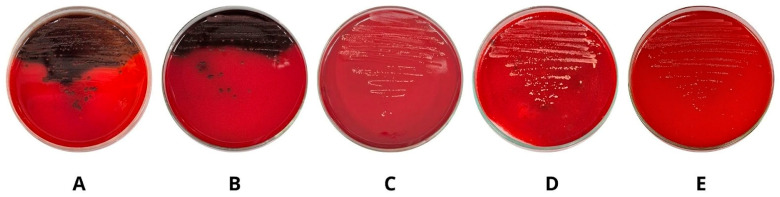
Effect of AgNPs and their association with bacterial cellulose hydrogel on biofilm and exopolysaccharide (EPS) matrix production by methicillin-resistant *Staphylococcus aureus* (MRSA C074), evaluated using the Congo red agar method. (**A**) Untreated MRSA C047 biofilm; (**B**) MRSA C047 biofilm after treatment with AgNP-A1000; (**C**) MRSA C047 biofilm after treatment with AgNP-C250; (**D**) MRSA C047 biofilm after treatment with HG-AgNP-A1000; (**E**) MRSA C047 biofilm after treatment with HG-AgNP-C250.

The MRSA C047 strain exhibited black-colored colonies with a dry appearance, associated with biofilm formation and the production of an extracellular polymeric substance (EPS) matrix, as observed during cultivation on Congo red agar (Figure 5A). After incorporation of AgNP-A1000 (Figure 5B), AgNP-C250 (Figure 5C), HG-AgNP-A1000 (Figure 5D), and HG-AgNP-C250 (Figure 5E) into the Congo red agar, a significant reduction in biofilm formation and EPS matrix production was observed, particularly when the bacteria were exposed to HG-AgNP-A1000 and HG-AgNP-C250.

### 2.6. Scanning Electron Microscopy of Antibiofilm Activity

**Figure 6:**
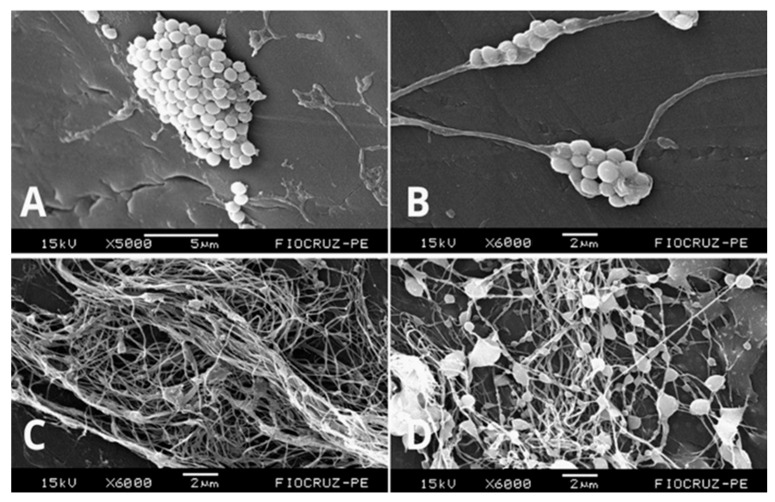
Scanning electron micrographs of the antibiofilm activity of silver nanoparticles against *Staphylococcus aureus*. (**A**) *Staphylococcus aureus* biofilm; (**B**) *Staphylococcus aureus* biofilm altered by the hydrogel; (**C**) AgNPs incorporated into the bacterial cellulose hydrogel; (**D**) AgNPs incorporated into the bacterial cellulose hydrogel against *Staphylococcus aureus*.

After treatment, SEM analysis was performed, showing biofilm formation by the MRSA ATCC 33591 strain (Figure 6A). Additionally, the effective incorporation of nanoparticles into the hydrogel (Figure 6C) and the hydrogel’s ability to interfere with biofilm formation through bacterial cell disaggregation (Figure 6B) were observed. Figure 6D shows complete biofilm degradation due to the action of silver nanoparticles incorporated into the hydrogel (HG-AgNP-C250) against *S. aureus*, demonstrating both antibacterial and antibiofilm potential via cell membrane damage and a reduction in exopolysaccharides (EPS) in the biofilm matrix.

## 3. Discussion

Size, shape, and surface charge are crucial factors influencing the biological effects of AgNPs. Regarding particle size, smaller particles exhibit an increased surface contact area, leading to the release of a greater amount of Ag^+^ ions [13]. In addition, the antimicrobial activity of AgNPs can be enhanced by chemical interactions resulting from direct contact between the nanoparticles and the bacterial cell surface, causing membrane damage and structural alterations that ultimately lead to cell death. Thus, isotropic geometries, such as those of the AgNPs evaluated in this study, demonstrate high efficacy due to their elevated surface-to-volume ratio, which provides greater reactivity [14].

Moreover, a positive surface charge promotes attraction to electronegative components of bacterial membranes. In this study, the AgNPs exhibited a positive charge, as demonstrated by the zeta potential, leading to disruption of the cellular membrane. This effect, combined with the release of silver ions, inhibits cellular metabolism and enhances antibacterial activity [15].

FTIR spectra (Figure 3) of chitosan (CH), AgNPs-A1000, and AgNPs-C250 show O–H and N–H stretching vibrations in the ~3400–3200 cm^−1^ range. For CH, the intense broad band arises from stretching vibrations of hydroxyl (O–H) and amine (N–H) groups. In contrast, the AgNPs exhibit a reduction in the intensity of this band, indicating interaction between the amino groups and the surface of the silver nanoparticles. This decrease is consistent with the formation of coordination bonds or weakened hydrogen bonding. The region around ~1650 cm^−1^ corresponds to the amide I band, in which CH shows a moderate peak, while both nanoparticle samples display a reduction in intensity or a possible peak shift, suggesting interaction between the amine groups and silver.

The ~1550 cm^−1^ region corresponds to the amide II band associated with N–H deformation vibrations, in which CH exhibits a significant peak, as expected due to this deformation, whereas the AgNPs show a reduction in intensity or a band shift associated with interaction with the nanoparticle surface. In the ~1400–1370 cm^−1^ range, C–H bending vibrations are observed, and the decreased intensity of this peak indicates interaction between chitosan and the silver surface. The ~1080–1030 cm^−1^ region corresponds to C–O–C and C–O stretching vibrations, and CH displays a sharp peak due to the presence of glycosidic bonds in the polymer, while in the AgNP spectra, a reduction or modification of this band is observed, suggesting electrostatic interactions with the nanoparticles. Additionally, in the region below 800 cm^−1^, the AgNP spectra exhibit the appearance of new peaks, which are typically indicative of the presence of metallic nanoparticles [16,17].

Most of the strains evaluated in this study showed sensitivity to AgNPs, both in their isolated form and when incorporated into the hydrogel, confirming their antibacterial potential against resistant Gram-positive bacteria, particularly species of the genus *Staphylococcus*. These findings are consistent with previous studies that also demonstrated the effectiveness of AgNPs against *S. aureus* and *S. epidermidis* [18,19]. Furthermore, it was observed that the bacterial cellulose hydrogel without AgNPs exhibited no antibacterial activity; however, it stands out as an efficient matrix for molecular incorporation and shows potential for wound-healing applications.

The time–kill kinetics of clinical MRSA and MRSE isolates were evaluated using nanoparticles incorporated into the hydrogel. Assessing antibacterial activity through this method is crucial for increasing the likelihood of more effective therapy, as it provides insight into the temporal efficacy of the antimicrobial agent. Moreover, the results of this assay can serve as a basis for in vivo studies, as they enable a deeper understanding of cell death kinetics and bacterial resistance within a population, thereby providing data for the development of more effective antimicrobial strategies [20]. Accordingly, Figure 4 shows that the activity of HG-AgNP-C250 and AgNP-C250 is similar and remains effective over time, indicating that the hydrogel does not interfere with the activity of the nanoparticles nor does it exhibit intrinsic antimicrobial activity. A subtle difference in antibacterial action time may reflect the controlled release of nanoparticles from the hydrogel matrix, highlighting the system as a promising vehicle for sustained silver. Nevertheless, the hydrogel plays a role in controlling the release and overall effect of the AgNPs [21].

As a virulence factor capable of promoting bacterial resistance in *Staphylococcus* spp., the ability to produce biofilms represents a major concern in the field of public health, since biofilms can be present on both biotic and abiotic surfaces, such as catheters used in hospitalized patients [22]. Biofilms constitute an adaptive survival mechanism in adverse environments, enabling bacteria to colonize a wide range of surfaces, including catheters and hospital equipment [23]. The results of this study corroborate previous reports demonstrating that the majority of strains belonging to the genus *Staphylococcus* produce biofilms at moderate to strong levels [22].

The antibiofilm potential of AgNPs has already been demonstrated in previous studies, as well as in the present investigation. In this context, AgNPs exhibited antibiofilm activity against Gram-positive bacteria, a finding that is corroborated by earlier reports [14,24]. The promising antibiofilm activity profile of AgNPs may be attributed to their enhanced intracellular penetration capacity, which can interfere with enzymatic and signal transduction pathways, altering multiple components of bacterial metabolism. In addition, AgNPs are able to penetrate through the pores and water channels of biofilms, which range from 50 to 500 nm in diameter, and their positively charged surface enables electrostatic interactions with the negatively charged biofilm matrix [10,25].

As observed in other studies, the isolated hydrogel did not exhibit antimicrobial activity, but it possesses antibiofilm potential, which can be enhanced when it serves as a carrier for a drug or a nanostructure [22]. The ability of these hydrogels to inhibit biofilm formation prevents bacterial–surface interactions, which occur through specific bonding, as the hydrogel induces surface modifications and also enables controlled release of the incorporated nanostructure [26,27]. Due to the reduction in bacterial–surface interactions induced by the hydrogel, decreased MBIC and MBEC values were observed for AgNPs when incorporated into the hydrogel, as it enhances their antibiofilm activity. The antibiofilm potential of hydrogels containing AgNPs or other drugs has been previously demonstrated, showing promising results against *Staphylococcus* biofilms [22,28].

HG-AgNP formulations demonstrated enhanced antibiofilm activity compared with free AgNPs against most of the strains evaluated, a finding previously reported in the literature and attributed to physicochemical modifications of the hydrogel network following nanosystem incorporation [29]. Incorporation of AgNPs into the hydrogel matrix has been shown to improve parameters such as the swelling index [30], thereby modulating the structural integrity and fluid uptake capacity of the polymeric network, key determinants of sustained antibiofilm performance. Furthermore, embedding AgNPs within the hydrogel enhances their diffusion and penetration into the biofilm structure, promoting increased interaction with the extracellular polymeric substances and contributing to improved antimicrobial efficacy [31].

Scanning electron microscopy confirmed the antibiofilm effect of AgNPs, both in their free form and when incorporated into the hydrogel, revealing cellular damage and biofilm reduction. A previous study demonstrated that hydrogels and biopolymers, such as bacterial cellulose, enhance the antimicrobial activity of nanoparticles, increasing their efficacy in controlling *Staphylococcus* biofilms [28].

The results of this study demonstrate the significant antibacterial potential of AgNPs against *S. aureus* and *S. epidermidis* strains, both in their free form and when incorporated into bacterial cellulose hydrogel. The matrix did not interfere with the efficacy of the nanoparticles, acting as a controlled release system and maintaining their activity over time. Although isolated bacterial cellulose does not exhibit antimicrobial activity, it supports regenerative capacity, accelerating wound healing, and its ability to modify surfaces and reduce bacterial adhesion contributed to enhanced antibiofilm activity when combined with AgNPs, resulting in decreased MBIC and MBEC values. Congo red assay and SEM analysis confirmed cellular damage and biofilm disruption under treated conditions, reinforcing the combined action.

The present study presents notable limitations, including the lack of cytotoxicity assessment of the developed material, antimicrobial testing restricted to Gram-positive strains, and the absence of in vivo validation. Therefore, further studies are needed to better understand and characterize the final formulation.

Thus, the incorporation of AgNPs with bacterial cellulose hydrogel emerges as a promising formulation for the treatment of skin infections, particularly those associated with biofilm formation by *S. aureus* and *S. epidermidis*.

## 4. Materials and Methods

### 4.1. Synthesis and Characterization of AgNPs and AgNPs-Loaded Hydrogel

Chitosan-stabilized silver nanoparticles (AgNPs) were synthesized via a seed-mediated growth approach according to the methodology described by Junior et al. [32]. Initially, AgNP seeds were prepared by adding an aqueous AgNO_3_ solution (1.5 mL, 0.11 mol L^−1^) to a chitosan solution (30 mL, 0.01 g mL^−1^) previously dissolved in 1% (*v*/*v*) acetic acid under continuous stirring in an ice-cooled bath. Subsequently, an aqueous NaBH_4_ solution (1.5 mL, 0.66 mol L^−1^) was added dropwise, resulting in an immediate color change to bright yellow, indicating the formation of chitosan-stabilized AgNP seeds. The agitation continued for 2 h. These seeds were removed from the ice bath and stored in the dark. For the growth step, an aqueous ascorbic acid solution (0.75 mL, 0.11 mol L^−1^) was added to a chitosan solution (30 mL, 0.01 g mL^−1^ in 1% acetic acid) under stirring, followed by the addition of different volumes (1 mL for A1000 and 0.250 mL for C250) of the freshly prepared AgNPs seed solution. Finally, an aqueous AgNO_3_ solution (0.40 mL, 0.11 mol L^−1^) was added dropwise under vigorous stirring at room temperature. The particle size and optical properties of the AgNPs were controlled by adjusting the amount of seed solution, leading to colloids with distinct colors ranging from yellow to orange and purple. The resulting chitosan-stabilized AgNP colloids were stored in the dark at room temperature. The total silver content in the AgNP dispersions was quantitatively determined by inductively coupled plasma optical emission spectroscopy (ICP-OES, Optima 7000 DV, Perkin Elmer, Shelton, CT, USA) after appropriate acid digestion of the samples (0.250 L) in aqueous acid solution (22.5 mL, 5% HNO_3_). The silver quantification was carried out three times and results were average.

The AgNPs were characterized in terms of particle size using dynamic light scattering (DLS) and surface charge by electrophoretic mobility, following methodologies established by our research group [25]. For morphological analysis, the nanoparticle formulations were diluted in ultrapure water (1:10 *v*/*v*), deposited on stubs, and kept in a desiccator at room temperature for 24 h. After drying, metallization was performed using a sputter coater (FINE COAT, Ikeja, Nigeria, ION SPUTTER JFC-1100), and the samples were examined under a transmission electron microscope (TEM) (FEI, Lausanne, Switzerland, Tecnai20 G2, 200 kV) [33].

For Fourier transform infrared spectroscopy (FTIR) analysis, spectra of the samples were obtained by mixing the lyophilized powders with potassium bromide (KBr) and analyzing them using a Fourier transform spectrophotometer. The samples were scanned from 4000 to 400 cm^−1^, and the spectra were recorded with a resolution of 4 cm^−1^ [33].

Field emission gun scanning electron microscopy (FEG-SEM) was used to determine the morphological characteristics of the hydrogel using a MIRA3 microscope (TESCAN, Brno, Czech Republic) operated at 15 kV acceleration voltage and a working distance of 10 mm, with a magnification of 100 k×. For these analyses, the samples were mounted on aluminum stubs and coated with a thin layer of gold before examination [22].

### 4.2. Incorporation of AgNPs into the Bacterial Cellulose Hydrogel

The bacterial cellulose biopolymer, an exopolysaccharide, was derived from sugarcane molasses [12]. It was produced by POLISA Biopolímeros para a Saúde Ltda (Indaiatuba, Brazil), start up at EECAC-UFRPE and sterilized by gamma ray at DEN-UFPE. For the incorporation of AgNPs, the previously prepared AgNPs were added dropwise in a 1:1 ratio under vigorous stirring at room temperature to the bacterial cellulose hydrogel (HG-CB) [11].

### 4.3. Evaluation of the Antibacterial Activity of AgNPs and AgNPs-Loaded Hydrogel

The antibacterial activity was evaluated by determining the minimum inhibitory concentration (MIC) using the broth microdilution method in accordance with the Clinical and Laboratory Standards Institute [34]. Initially, Müeller–Hinton broth (MHB) was added to microdilution plates. The test products, AgNP-A1000, AgNP-C250, and the AgNPs-loaded hydrogel (HG-AgNP-A1000 and HG-AgNP-C250), were then added by serial dilution. Clinical biofilm-forming strains of MRSA and MRSE were used. Bacterial suspensions were adjusted to a 0.5 McFarland standard and deposited into the wells. The plates were incubated at 35 ± 2 °C for 24 h, and absorbance readings were taken at 620 nm using a spectrophotometer. The MIC was defined as the lowest concentration capable of inhibiting more than 90% of microbial growth. The minimum bactericidal concentration (MBC) was determined following the MIC results. An aliquot from wells showing no growth was inoculated onto Müeller–Hinton agar (MHA) plates, which were then incubated at 35 ± 2 °C for 24 h. After incubation, the MBC was defined as the lowest concentration at which no microbial growth was observed [34].

### 4.4. Time–Kill Assay

The bacterial time–kill curve after treatment with AgNP-C250 and HG-AgNP-C250 was performed using the procedure described by Correia [22]. The time–kill curves were determined using concentrations corresponding to ½ MIC. *S. aureus* and *S. epidermidis* strains were inoculated onto MHA and incubated at 35 ± 2 °C for 24 h. After this period, the bacterial suspension was adjusted to a concentration of 1.0 × 10^6^ CFU/mL. The samples were incubated at 35 ± 2 °C for 24 h, and aliquots were withdrawn at 0, 3, 6, 9, 12, and 24 h, then plated on MHA using the pour-plate technique after serial dilutions in sterile saline solution. The plates were incubated at 35 ± 2 °C for 24 h. Results were expressed as log_10_ colony-forming units per milliliter (log_10_ CFU/mL). Bactericidal activity was defined as a reduction of ≥3 log_10_ CFU/mL, corresponding to a 99.9% inhibition of bacterial growth, while bacteriostatic activity was determined as a reduction of ≤2 log_10_ CFU/mL compared to the control growth curve [22].

### 4.5. Determination of Biofilm Inhibition

The determination of the minimum biofilm inhibitory concentration (MBIC) was performed according to Correia [22]. The bacterial strains used were biofilm producers, showing moderate to strong biofilm formation, as described in the studies by Correia [22] and Campos [25]. Initially, Tryptone Soy Broth (TSB) supplemented with glucose was added to each well of the microdilution plates, followed by serial dilutions of AgNP-A1000, AgNP-C250, HG-AgNP-A1000, and HG-AgNP-C250, and finally, the bacterial suspensions. The microplates were incubated at 35 °C for 24 h. After incubation, the contents of the wells were aspirated, and the wells were washed with phosphate buffer (pH 7.4). The plates were then dried, and the adhered bacteria were fixed with 99% methanol. After fixation, the methanol was removed, and the plates were allowed to dry again. Subsequently, the adhered bacteria were stained with 1% crystal violet. The results were analyzed using a spectrophotometer at 570 nm. The MBIC was defined as the lowest concentration capable of inhibiting biofilm formation [22].

### 4.6. Evaluation of the Antibiofilm Activity by the Congo Red Agar Method

The qualitative determination of biofilm and exopolysaccharide (EPS) matrix inhibition was performed using the Congo red method, according to Correia [22]. The MRSA C047 strain was adjusted in brain heart infusion (BHI) broth to 0.5 on the McFarland scale and incubated at 35 ± 2 °C for 24 h. An aliquot of the bacterial culture was then plated on Petri dishes containing Congo red agar supplemented with AgNP-A1000, AgNP-C250, HG-AgNP-A1000, and HG-AgNP-C250 at their respective MBICs. The plates were incubated at 35 ± 2 °C for 48 h. After the incubation period, colonies with black coloration and rough consistency were considered biofilm producers, while red colonies represented bacteria that did not produce biofilm. Independent experiments were performed in duplicate on different days.

### 4.7. Determination of Biofilm Eradication

The determination of the minimum biofilm eradication concentration (MBEC) was performed according to Correia [22]. Initially, bacterial inocula were adjusted to a 0.5 McFarland standard in TSB supplemented with glucose and distributed into microdilution plates containing all strains that exhibited moderate to strong biofilm production. The plates were incubated at 35 ± 2 °C for 24 h to allow biofilm formation. After incubation, the culture medium was removed and replaced with fresh medium. Subsequently, AgNP-A1000, AgNP-C250, HG-AgNP-A1000, and HG-AgNP-C250 were added to the microplates through serial dilution, and the plates were incubated again at 35 ± 2 °C for 24 h. After incubation, biofilm quantification was performed using the crystal violet method, as previously described. The MBEC was defined as the lowest concentration capable of eradicating the formed biofilm [22].

### 4.8. Scanning Electron Microscopy of Antibiofilm Activity

Initially, MRSA ATCC 33591 suspensions were adjusted to 0.5 on the McFarland scale and exposed to HG-AgNP-C250 treatments at the MBIC. Bacteria not exposed to any treatment were used as positive controls for biofilm formation. The samples were prepared using 14G catheters, placed in the wells of a flat-bottom 24-well plate, and incubated for 24 h at 35 ± 2 °C. After incubation, the samples were washed three times with PBS buffer and fixed in a solution containing 2.5% glutaraldehyde and 4% paraformaldehyde in 0.1 M cacodylate buffer (pH 7.2) for 12 h. Subsequently, 1% osmium tetroxide (OsO_4_) was added for post-fixation, followed by dehydration through a graded ethanol series. After dehydration, the samples were subjected to critical point drying using liquid CO_2_ and sputter-coated with gold. The prepared samples were examined using a scanning electron microscope (SEM; Jeol JSM-5600, Tokyo, Japan) operated at 15 kV. All experiments were performed in independent triplicates on different days to ensure reproducibility of the results [22].

## 5. Conclusions

The characterization of AgNPs revealed suitable sizes, morphologies, and surface charges for antibacterial activity. The hydrogel exhibited a three-dimensional network favorable for nanoparticle incorporation, enhancing its potential application. The antibacterial effect of the HG-AgNPs was confirmed and showed promising results. The time–kill curve studies demonstrated that bacterial growth was inhibited after treatment with AgNP-C250 at 6 h and HG-AgNP-C250 at 9 h. In the antibiofilm activity assays, the HG-AgNPs showed greater efficiency both in inhibiting biofilm formation and in eradicating established biofilms, as evidenced by scanning electron microscopy. Overall, these findings indicate that HG-AgNP-A1000 and HG-AgNP-C250 represent a promising therapeutic approach for the treatment of skin and wound infections caused by resistant strains of *Staphylococcus aureus* and *Staphylococcus epidermidis*. However, as they are based exclusively on in vitro assays, these findings require additional investigations into cytocompatibility and silver release kinetics to support their clinical applicability.

## Figures and Tables

**Table 1:** Characterization of absorption spectrum, particle size, PDI and zeta potential of AgNPs.

Sample	λ Max (nm)	Ø (nm)	PDI	Zeta Potential (mV)
A1000	431	35 ± 9.07	0.29 ± 0.01	+36.56 ± 1.29
C250	478	59 ± 11.05	0.30 ± 0.02	+32.75 ± 1.84

**Table 2:** Antibacterial activity of AgNP-A1000, AgNP-C250, HG-AgNP-A1000, and HG-AgNP-C250 against *Staphylococcus aureus* strains.

Strains	AgNP-A1000		AgNP-C250		HG-AgNP-A1000		HG-AgNP-C250	
	MIC	MBC	MIC	MBC	MIC	MBC	MIC	MBC
	μg/mL							
MRSA ATCC 33591	50	50	50	50	50	50	50	50
MRSA C047	25	50	25	50	25	50	25	50
MRSA C074	25	50	25	50	25	50	25	50
MRSA C128	25	50	25	50	25	50	25	50
MRSA C137	50	50	50	50	50	50	50	50

MIC: minimum inhibitory concentration; MBC: minimum bactericidal concentration; MRSA: methicillin-resistant *Staphylococcus aureus*; ATCC: American Type Culture Collection; C047, C074, C128, and C137: clinical isolates of MRSA; AgNP-A1000: silver nanoparticles; AgNP-C250: silver nanoparticles; HG-AgNP-A1000: hydrogel containing AgNP-A1000; HG-AgNP-C250: hydrogel containing AgNP-C250.

**Table 3:** Antibacterial activity of AgNP-A1000, AgNP-C250, HG-AgNP-A1000, and HG-AgNP-C250 against *Staphylococcus epidermidis* strains.

Strains	AgNP-A1000		AgNP-C250		HG-AgNP-A1000		HG-AgNP-C250	
	MIC	MBC	MIC	MBC	MIC	MBC	MIC	MBC
	μg/mL							
*S. epidermidis* ATCC 12228	12.5	25	12.5	25	25	50	25	50
MRSE C233	12.5	25	12.5	25	12.5	25	12.5	25
MRSE C266	6.25	25	6.25	25	6.25	25	6.25	25
MRSE C271	25	50	25	50	50	50	50	50
MRSE C281	12.5	25	12.5	25	12.5	25	12.5	25

MIC: minimum inhibitory concentration; MBC: minimum bactericidal concentration; MRSE: methicillin-resistant *Staphylococcus epidermidis*; ATCC: American Type Culture Collection; C233, C266, C271, and C281: clinical isolates of MRSE; AgNP-A1000: silver nanoparticles; AgNP-C250: silver nanoparticles; HG-AgNP-A1000: hydrogel containing AgNP-A1000; HG-AgNP-C250: hydrogel containing AgNP-C250.

**Table 4:** Antibiofilm activity of AgNP-A1000, AgNP-C250, HG-AgNP-A1000, and HG-AgNP-C250 against *Staphylococcus aureus* strains.

Strains	AgNP-A1000		AgNP-C250		HG-AgNP-A1000		HG-AgNP-C250	
	MBIC	MBEC	MBIC	MBEC	MBIC	MBEC	MBIC	MBEC
	μg/mL							
MRSA ATCC 33591	1.56	100	1.56	50	6.25	>100	1.56	50
MRSA C047	3.125	>100	25	6.25	25	>100	12.5	50
MRSA C074	25	50	25	25	25	50	6.25	12.5
MRSA C128	25	3.125	12.5	50	25	25	12.5	12.5
MRSA C137	50	25	25	25	25	25	12.5	1.56

MBIC: minimum biofilm inhibitory concentration; MBEC: minimum biofilm eradication concentration; MRSA: Methicillin-resistant *Staphylococcus aureus*; ATCC: American Type Culture Collection; C047, C074, C128, and C137: clinical isolates of MRSA; AgNP-A1000: silver nanoparticles; AgNP-C250: silver nanoparticles; HG-AgNP-A1000: hydrogel containing AgNP-A1000; HG-AgNP-C250: hydrogel containing AgNP-C250.

**Table 5:** Antibiofilm activity of AgNP-A1000, AgNP-C250, HG-AgNP-A1000, and HG-AgNP-C250 against *Staphylococcus epidermidis* strains.

Strains	AgNP-A1000		AgNP-C250		HG-AgNP-A1000		HG-AgNP-C250	
	MBIC	MBEC	MBIC	MBEC	MBIC	MBEC	MBIC	MBEC
	μg/mL							
*S. epidermidis* ATCC 12228	12.5	25	1.56	25	25	50	6.25	25
MRSE C233	12.5	12.5	3.125	3.125	12.5	25	3.125	25
MRSE C266	6.25	6.25	3.125	12.5	6.25	25	3.125	50
MRSE C271	25	50	3.125	12.5	50	50	3.125	12.5
MRSE C281	6.25	12.5	0.78	6.25	12.5	25	6.25	25

MBIC: minimum biofilm inhibitory concentration; MBEC: minimum biofilm eradication concentration; MRSE: Methicillin-resistant *Staphylococcus epidermidis*; ATCC: American Type Culture Collection; C233, C266, C271 and C281: clinical isolates of MRSE; AgNP-A1000: silver nanoparticles; AgNP-C250: silver nanoparticles; HG-AgNP-A1000: hydrogel containing AgNP-A1000; HG-AgNP-C250: hydrogel containing AgNP-C250.

## Data Availability

The original contributions presented in this study are included in the article. Further inquiries can be directed to the corresponding authors.
